# Atomistic simulations of out-of-equilibrium quantum nuclear dynamics

**DOI:** 10.1038/s41524-025-01588-4

**Published:** 2025-04-16

**Authors:** Francesco Libbi, Anders Johansson, Lorenzo Monacelli, Boris Kozinsky

**Affiliations:** 1 John A. Paulson School of Engineering and Applied Sciences, Harvard University, Cambridge, MA 02138 USA; 2 Department of Physics, Sapienza University of Rome, Piazzale Aldo Moro 5, 00185 Rome, Italy; 3 Robert Bosch LLC Research and Technology Center, Watertown, MA 02472 USA

**Keywords:** Computational methods, Ferroelectrics and multiferroics

## Abstract

The rapid advancements in ultrafast laser technology have paved the way for pumping and probing the out-of-equilibrium dynamics of nuclei in crystals. However, interpreting these experiments is extremely challenging due to the complex nonlinear responses in systems where lattice excitations interact, particularly in crystals composed of light atoms or at low temperatures where the quantum nature of ions becomes significant. In this work, we address the nonequilibrium quantum ionic dynamics from first principles. Our approach is general and can be applied to simulate any crystal, in combination with a first-principles treatment of electrons or external machine-learning potentials. It is implemented by leveraging the nonequilibrium time-dependent self-consistent harmonic approximation (TD-SCHA), with a stable, energy-conserving, correlated stochastic integration scheme that achieves an accuracy of ${\mathcal{O}}(d{t}^{3})$. We benchmark the method with both a simple one-dimensional model to test its accuracy and a realistic 40-atom cell of SrTiO_3_ under THz laser pump, paving the way for simulations of ultrafast THz-Xray pump-probe spectroscopy like those performed in synchrotron facilities.

## Introduction

Nuclear quantum effects often play a crucial role in determining properties of materials^1^, affecting their thermodynamic stability^2–4^, electronic structure^5,6^, and transport phenomena^7^. Understanding and accurately accounting for quantum contributions in tunneling and vibrational statistics requires simulations that go beyond classical nuclei approximation. Path-integral molecular dynamics (PIMD) is the most common approach to simulate nuclear quantum effects in complex anharmonic crystals^8–11^, especially with the ring-polymer formulation^12,13^, which is an exact framework for sampling the equilibrium nuclear density matrix. However, PIMD is rigorously formulated with an assumption of thermodynamic equilibrium as it evolves the trajectories in imaginary time. Simulating out-of-equilibrium quantum nuclear dynamics requires a theory for the real-time evolution, such as the path-integral quantum Monte Carlo (PIMC)^14–17^, which is extremely challenging due to the appearance of the so-called sign problem, in which different trajectories contribute with different signs, introducing noise in determining dynamical observables^18^. Therefore, most applications of real-time PIMC remain limited to systems composed of only a few degrees of freedom^19^.

Several techniques have been proposed to overcome the limits of real-time PIMC to study nuclear quantum effects in systems of realistic interest^20–22^. Among these, the time-dependent self-consistent harmonic approximation (TD-SCHA^23,24^) holds significant promise. The theory extends the stochastic self-consistent harmonic approximation (SSCHA)^25–27^, a well-established technique to simulate equilibrium thermodynamics of solids accounting for quantum nuclear fluctuations. The success of SSCHA lies in the adoption of approximations that are particularly effective for crystals, achieving a computational cost that is orders of magnitude lower than PIMD, while still producing predictions in very good agreement with experiments^2,3,7,28,29^.

TD-SCHA has already been employed in the linear response regime, where it enabled the prediction of Raman and IR spectra of metallic hydrogen with unprecedented accuracy^30^. However, up to now, no application of TD-SCHA beyond equilibrium has been attempted. The difficulty in obtaining accurate and stable dynamic solutions of TD-SCHA equations is challenging due to the need for the evaluation of ensemble averages of the nuclear potential energy landscape.

In this work we address this challenge by developing an algorithm to solve the dynamical TD-SCHA equations and simulate the out-of-equilibrium dynamics of nuclei in complex realistic systems. In Section “Time-dependent self-consistent harmonicapproximation”, we revise the TD-SCHA equations of motion for the nuclear density matrix. Section “Numerical integration of TD-SCHA equations” introduces three different numerical algorithms to integrate the TD-SCHA equations, and their stability is discussed in Section “Stability of the integration schemes”. Section “Stochastic formulation” presents the correlated sampling technique to perform the evaluation of the stochastic quantum averages. This formulation ensures both the efficient evaluation of ensemble averages and the numerical stability of the equations. Crucially, we show that such a correlated approach conserves energy in one dimensional problems independently of the number of stochastic configurations adopted. We benchmark the method in Section “Tests”, where we compare the different numerical schemes on a one dimensional model system. Finally, in Section “Dynamics in SrTiO_3_”, we provide an example of the application of TD-SCHA to realistic systems by studying the quantum dynamics in SrTiO_3_ (STO) when driven out of equilibrium by a strong laser pulse of THz frequency.

## Results and discussion

### Time-dependent self-consistent harmonic approximation

The TD-SCHA formulation leverages the Wigner formalism^31,32^. The Wigner transform of the nuclear quantum density matrix $\hat{\rho }(t)$ describing the quantum state is defined as

1 $$\rho ({\bf{R}},{\bf{P}},t)=\int\frac{{e}^{-\frac{i}{\hslash }{\bf{P}}\cdot {{\bf{R}}}^{{\prime} }}}{{(2\pi \hslash )}^{3N}}\left\langle {\bf{R}}+\frac{{{\bf{R}}}^{{\prime} }}{2}| \hat{\rho }(t)| {\bf{R}}-\frac{{{\bf{R}}}^{{\prime} }}{2}\right\rangle d{{\bf{R}}}^{{\prime} },$$

and it maps the quantum operator $\hat{\rho }(t)$ into a function of positions and momenta *ρ*(**R**, **P**, *t*) that is analogous to the classical nuclear density. All atomic quantities are rescaled by mass to simplify the notation: ${R}_{i}={\tilde{R}}_{i}\sqrt{{m}_{i}}$ and ${P}_{i}={\tilde{P}}_{i}/\sqrt{{m}_{i}}$, where the index *i* goes from 1 to 3*N*, containing both the Cartesian and atomic index, and the tilde indicates the standard (not mass-rescaled) quantities. Dynamical averages of any quantum observable are obtained by tracing **R** and **P** on the density:

2 $$\langle O \rangle (t)=\int\,d{\bf{R}}d{\bf{P}}\rho ({\bf{R}},{\bf{P}},t)O({\bf{R}},{\bf{P}}).$$

The TD-SCHA method is based on expressing the Wigner density matrix as a general Gaussian form in terms of ionic positions **R** and momenta **P**:

3 $$\rho ({\bf{R}},{\bf{P}},t)=\frac{1}{{\mathcal{N}}}{e}^{-\frac{1}{2}(\delta {\bf{R}}\cdot {\boldsymbol{\alpha }}\cdot \delta {\bf{R}}+\delta {\bf{P}}\cdot {\boldsymbol{\beta }}\cdot \delta {\bf{P}}+\delta {\bf{R}}\cdot {\boldsymbol{\gamma }}\cdot \delta {\bf{P}})}\,.$$

Here $\delta {\bf{R}}(t)={\bf{R}}-{\bf{\mathcal{R}}}(t)$ and $\delta {\bf{P}}(t)={\bf{P}}-{\bf{\mathcal{P}}}(t)$ and ${\mathcal{N}}$ is the normalization factor, where ${\bf{\mathcal{R}}}(t)=\left\langle {\bf{R}}\right\rangle (t)$ represent average positions and ${\bf{\mathcal{P}}}(t)=\left\langle {\bf{P}}\right\rangle (t)$ the average momenta. The ***α***(*t*), ***β***(*t*) and ***γ***(*t*) matrices are related to, respectively, position-position, momentum-momentum, and position-momentum covariances by the following relations:

4 $${{\bf{A}}}^{-1}={\left\langle \delta {R}_{i}\delta {R}_{j}\right\rangle }^{-1}={\boldsymbol{\alpha }}-{\boldsymbol{\gamma }}\cdot {{\boldsymbol{\beta }}}^{-1}\cdot {{\boldsymbol{\gamma }}}^{T},$$

5 $${{\bf{B}}}^{-1}={\left\langle \delta {P}_{i}\delta {P}_{j}\right\rangle }^{-1}=-{{\boldsymbol{\gamma }}}^{T}+{\boldsymbol{\beta }}\cdot {{\boldsymbol{\gamma }}}^{-1}\cdot {\boldsymbol{\alpha }}\,,$$

6 $${{\mathbf{\Gamma }}}^{-1}={\left\langle \delta {R}_{i}\delta {P}_{j}\right\rangle }^{-1}={\boldsymbol{\beta }}-{{\boldsymbol{\gamma }}}^{T}\cdot {{\boldsymbol{\alpha }}}^{-1}\cdot {{\boldsymbol{\gamma }}}^{T}\,.$$

The evolution of the density Wigner-space density matrix *ρ*(**R**, **P**, *t*) is determined by the propagation in time of the parameters ${\bf{\mathcal{R}}}(t)$, ${\bf{\mathcal{P}}}(t)$, **A**(*t*), **B**(*t*) and **Γ**(*t*). Analogously to the time-dependent Hartree–Fock or time-dependent density functional theory for electrons, the time evolution is obtained by imposing the least action principle^24^, leading to the time-dependent self-consistent Liouville-von Neumann equation for the density matrix:

7 $$i\hslash \frac{\partial \hat{\rho }}{\partial t}=\left[{\mathcal{H}}[\hat{\rho }],\hat{\rho }\right],$$

where ${\mathcal{H}}[\hat{\rho }]$ is a self-consistent harmonic Hamiltonian whose parameters depend on the anharmonic potential and the density matrix $\hat{\rho }$ and the square brackets indicate the quantum commutator (more details in ref. ^23^). Notably, in TD-SCHA, ${\mathcal{H}}[\hat{\rho }]$ is local in time, so the time evolution depends only on the current quantum state. Expressing Eq. (7) in the Wigner formalism and substituting the Gaussian form for the density matrix leads to the set of differential equations

8 $$\left\{\begin{array}{ll}{\dot{\bf{\mathcal{R}}}}={\bf{\mathcal{P}}}\quad \\ {\dot{\bf{\mathcal{P}}}}=\left\langle {\bf{f}}\right\rangle \quad \\ {\dot{\bf{A}}}={\mathbf{\Gamma }}+{{\mathbf{\Gamma }}}^{\dagger }\quad \\ {\dot{\bf{B}}}=-\langle {{\boldsymbol{\partial}}}^{2}V\rangle {\mathbf{\Gamma}}-{{\mathbf{\Gamma}}}^{\dagger }\langle {{\boldsymbol{\partial}}}^{2}V\rangle \quad \\ \dot{{\mathbf{\Gamma }}}={\bf{B}}-{\bf{A}}\langle {{\boldsymbol{\partial}}}^{2}V\rangle \quad \end{array}\right.\,,$$

where the dot over a tensor $\dot{\circ}$ indicates the time-derivative, the product between tensors is the standard rows-by-columns contraction among all indices, and the dagger symbol indicates the matrix transposition operation ${O}_{ij}^{\dagger }={O}_{ji}$. Here, the atomic potential energy landscape (PES), *V*(**R**, *t*), enters the quantum averages of forces 〈**f**〉 and the average curvature tensor 〈***∂***^2^*V*〉, defined as

9 $$\langle {f}_{a}\rangle (t)=-\int\,d{\bf{R}}d{\bf{P}}\frac{\partial V}{\partial {R}_{a}}(t)\rho ({\bf{R}},{\bf{P}},t),$$

10 $$\langle {\partial }_{ab}^{2}V\rangle (t)=\int\,d{\bf{R}}d{\bf{P}}\frac{{\partial }^{2}V}{\partial {R}_{a}\partial {R}_{b}}(t)\rho ({\bf{R}},{\bf{P}},t).$$

The solution of Eq. (8) provides the quantum state *ρ*(**R**, **P**, *t*), enabling the direct computation of the time envelope of any quantum observable.

The stationary solution of Eq. (8) coincides with the equilibrium fixed-volume state that minimizes the Helmholtz free energy and can be obtained with the standard SSCHA algorithm^24,25^.

When simulating a pump-probe experiment, the system is prepared at equilibrium and perturbed with a radiation pulse modeled as a time-dependent external potential *V*_ext_(**R**, *t*). The overall potential that enters in Eqs. (9) and (10) is

11 $$V({\bf{R}},t)={V}_{{\rm{BO}}}({\bf{R}})+{V}_{{\rm{ext}}}({\bf{R}},t),$$

where *V*_BO_(**R**) is the instantaneous interaction potential of nuclei within the Born-Oppenheimer approximation that depends only on the nuclear positions.

### Numerical integration of TD-SCHA equations

Numerical solutions of the TD-SCHA equations of motion (8) have so far been limited to simple one-dimensional models^24^ and linear-response calculations, enabled by an efficient Lanczos algorithm^23^. The major challenge to applying TD-SCHA in the out-of-equilibrium regime is associated with the cost of calculating the averages 〈**f**〉 and particularly 〈***∂***^2^*V*〉, Eq. (10), as sampling the second derivatives of the potential is computationally expensive. This section introduces a finite-difference scheme to integrate the TD-SCHA equations with an error scaling as ${\mathcal{O}}(d{t}^{3})$ that requires the computation of Eq. (10) only once per time step. Expanding the time evolution of TD-SCHA quantities in the Taylor series to second order in the time step increment *dt*, we derive the following expressions:

12 $$\left\{\begin{array}{ll}{{\bf{\mathcal{R}}}}_{t+dt}={{\bf{\mathcal{R}}}}_{t}+{{\bf{\mathcal{P}}}}_{t}dt+\frac{1}{2}\left\langle {{\bf{f}}}_{t}\right\rangle d{t}^{2}+{\mathcal{O}}(d{t}^{3})\quad \\ {{\bf{\mathcal{P}}}}_{t+dt}={{\bf{\mathcal{P}}}}_{t}+\left\langle {{\bf{f}}}_{t}\right\rangle dt+\frac{1}{2}{{\bf{\mathcal{P}}}}_{t}^{{\prime}{\prime}}d{t}^{2}+{\mathcal{O}}(d{t}^{3})\quad \\ {{\bf{A}}}_{t+dt}={{\bf{A}}}_{t}+{\left({\mathbf{\Gamma }}+{{\mathbf{\Gamma }}}^{\dagger }\right)}_{t}dt+\frac{1}{2}{\left({\bf{B}}-{\bf{A}}\langle {{\boldsymbol{\partial }}}^{2}V\rangle \right)}_{t}d{t}^{2}+\frac{1}{2}{\left({\bf{B}}-\langle {{\boldsymbol{\partial}}}^{2}V\rangle {\bf{A}}\right)}_{t}d{t}^{2}+{\mathcal{O}}(d{t}^{3})\quad \\ {{\bf{B}}}_{t+dt}={{\bf{B}}}_{t}-{\left(\langle {{\boldsymbol{\partial}}}^{2}V\rangle {\mathbf{\Gamma }}+{{\mathbf{\Gamma }}}^{\dagger }\langle {{\boldsymbol{\partial}}}^{2}V\rangle \right)}_{t}dt+\frac{1}{2}{{\bf{B}}}_{t}^{{\prime}{\prime}}d{t}^{2}+{\mathcal{O}}(d{t}^{3})\quad \\ {{\mathbf{\Gamma }}}_{t+dt}={{\mathbf{\Gamma }}}_{t}+{\left({\bf{B}}-{\bf{A}}\langle {{\boldsymbol{\partial}}}^{2}V\rangle \right)}_{t}dt+\frac{1}{2}{{\mathbf{\Gamma }}}_{t}^{{\prime}{\prime}}d{t}^{2}+{\mathcal{O}}(d{t}^{3})\quad \end{array}\right.$$

Notably, while the equations for the evolution of ${\bf{\mathcal{R}}}$ and **A** are explicit up to ${\mathcal{O}}(d{t}^{3})$, we need the values of the second derivatives of **B**_*t*_, **Γ**_*t*_ and ${{\bf{\mathcal{P}}}}_{t}$.

To preserve the ${\mathcal{O}}(d{t}^{3})$ error of the time propagation we use the central difference formula to approximate those second derivatives (see Supplementary Section II of the Supplementary Information, SI^33^):

13 $${F}_{t+dt}={F}_{t}+\frac{1}{2}({F}_{t}^{{\prime} }+{F}_{t+dt}^{{\prime} })dt+{\mathcal{O}}(d{t}^{3})\,,$$

where *F* represents a generic variable. This expression only requires the knowledge of its first derivatives at times *t* and *t* + *dt*, but not of the second derivative. We can rely on two observations: (i) The calculation of 〈**f**〉 and 〈***∂***^2^*V*〉 depends only on ${\bf{\mathcal{R}}}$ and **A** as the potential is a function of only the positions (see Eqs. (9), (10) and Supplementary Section I of the SI); (ii) both ${\bf{\mathcal{R}}}$ and **A** can be integrated explicitly with accuracy ${\mathcal{O}}(d{t}^{3})$. The Generalized Verlet (GV) algorithm we devise comprises the following steps: (I) calculate ${\bf{\mathcal{R}}}_{t+dt}$ and **A**_*t*+*dt*_ with accuracy ${\mathcal{O}}(d{t}^{3})$ using the first and the third of Eqs. (12) respectively; (II) use ${{\bf{\mathcal{R}}}}_{t+dt}$ and **A**_*t*+*dt*_ to determine ${\left\langle {\bf{f}}\right\rangle }_{t+dt}$ and ${\langle {{\boldsymbol{\partial }}}^{2}V\rangle }_{t+dt}$; (III) determine ${\bf{\mathcal{P}}}$, **B** and **Γ** using Eq. (13)

14 $$\left\{\begin{array}{ll}{{\bf{\mathcal{P}}}}_{t+dt}={{\bf{\mathcal{P}}}}_{t}+{\langle {\bf{f}}\rangle }_{\bar{t}}\,dt+{\mathcal{O}}({t}^{3})\quad \\ {{\bf{B}}}_{t+dt}={{\bf{B}}}_{t}-{\left(\langle {{\boldsymbol{\partial }}}^{2}V\rangle {\mathbf{\Gamma }}+{{\mathbf{\Gamma }}}^{\dagger }\langle {{\boldsymbol{\partial }}}^{2}V\rangle \right)}_{\bar{t}}dt+{\mathcal{O}}(d{t}^{3})\quad \\ {{\mathbf{\Gamma }}}_{t+dt}={{\mathbf{\Gamma }}}_{t}+{\left({\bf{B}}-{\bf{A}}\langle {{\boldsymbol{\partial }}}^{2}V\rangle \right)}_{\bar{t}}dt+{\mathcal{O}}(d{t}^{3})\,.\quad \end{array}\right.$$

Here we use the shorthand ${F}_{\bar{t}}$ to indicate $\frac{1}{2}({F}_{t}+{F}_{t+dt})$. Even though Eq. (14) is implicit in the variables **B** and **Γ**, it requires the calculation of 〈**f**〉 and 〈***∂***^2^*V*〉 just once per time step (due to observation (i)), and it evolves the parameters with accuracy ${\mathcal{O}}(d{t}^{3})$. Furthermore, the equations for integrating ${\bf{\mathcal{R}}}$ and ${\bf{\mathcal{P}}}$ coincide with the familiar *velocity Verlet* scheme^34^.

We now compare the above GV integration scheme with alternatives, namely explicit Euler (EE) and semi-implicit Euler (SIE) algorithms, showing that the GV is more accurate than both EE and SIE and stable for larger values of the time-step *dt*. Alternative integration strategies for Eqs. (12) are founded on the semi-implicit Euler method. Since **Γ** is related to the derivatives of **A** and **B** (see Eqs. (8)), the semi-implicit Euler (SIE) scheme consists in updating **Γ** first, then computing **A**_*t*+*dt*_ and **B**_*t*+*dt*_ by using **Γ**_*t*+*dt*_ instead of **Γ**_*t*_:

15 $$\left\{\begin{array}{ll}{{\bf{A}}}_{t+dt}={{\bf{A}}}_{t}+{\left({\mathbf{\Gamma }}+{{\mathbf{\Gamma }}}^{\dagger }\right)}_{t+dt}dt+{\mathcal{O}}(d{t}^{2})\quad \\ {{\bf{B}}}_{t+dt}={{\bf{B}}}_{t}-\left({\langle {{\boldsymbol{\partial }}}^{2}V\rangle }_{t}{{\mathbf{\Gamma }}}_{t+dt}+{{\mathbf{\Gamma }}}_{t+dt}^{\dagger }{\langle {{\boldsymbol{\partial }}}^{2}V\rangle }_{t}\right)dt+{\mathcal{O}}(d{t}^{2})\quad \\ {{\mathbf{\Gamma }}}_{t+dt}={{\mathbf{\Gamma }}}_{t}+{\left({\bf{B}}-{\bf{A}}\langle {{\boldsymbol{\partial }}}^{2}V\rangle \right)}_{t}dt+{\mathcal{O}}(d{t}^{2})\end{array}\right.\,.$$

Instead, ${\bf{\mathcal{R}}}$ and ${\bf{\mathcal{P}}}$ are evolved according to Verlet. These integration schemes can be compared to the simplest approach, the explicit Euler (EE) scheme, where all the parameters are evolved simultaneously:

16 $$\left\{\begin{array}{ll}{{\bf{A}}}_{t+dt}={{\bf{A}}}_{t}+{\left({\mathbf{\Gamma }}+{{\mathbf{\Gamma }}}^{\dagger }\right)}_{t}dt+{\mathcal{O}}(d{t}^{2})\quad \\ {{\bf{B}}}_{t+dt}={{\bf{B}}}_{t}-\left({\langle {{\boldsymbol{\partial }}}^{2}V\rangle }_{t}{{\mathbf{\Gamma }}}_{t}+{{\mathbf{\Gamma }}}_{t}^{\dagger }{\langle {{\boldsymbol{\partial }}}^{2}V\rangle }_{t}\right)dt+{\mathcal{O}}(d{t}^{2})\quad \\ {{\mathbf{\Gamma }}}_{t+dt}={{\mathbf{\Gamma }}}_{t}+{\left({\bf{B}}-{\bf{A}}\langle {{\boldsymbol{\partial }}}^{2}V\rangle \right)}_{t}dt+{\mathcal{O}}(d{t}^{2})\end{array}\right.\,.$$

### Stability of the integration schemes

Here, we investigate the stability of the integration schemes introduced in the previous section. Particularly, we are interested in the dynamics of the variables **A,** **B**, and **Γ**, which are not present in classical Newtonian nuclear dynamics equations.

Let us consider a 1D wave packet evolving in a Harmonic potential. Thanks to the constant curvature ***κ*** of the PES, **A,** **B** and **Γ** do not depend on the average coordinates (centroids) ${\bf{\mathcal{R}}}$ and ${\bf{\mathcal{P}}}$ since

17 $$\langle {{\boldsymbol{\partial }}}^{2}V\rangle ={\boldsymbol{\kappa }}.$$

The TD-SCHA equations for these variables reduce to

18 $$\left(\begin{array}{r} \dot{A}\\ \dot{B}\\ \dot{\Gamma}\end{array}\right)=\left(\begin{array}{rcl}0&0&2\\ 0&0&-2\kappa \\ -\kappa &1&0\end{array}\right)\left(\begin{array}{r}A\\ B\\ \Gamma \end{array}\right)\,.$$

By rescaling the parameters as

19 $$\left\{\begin{array}{l}{A}^{{\prime} }=\sqrt{\frac{\kappa }{2}}A\quad \\ {B}^{{\prime} }=\frac{1}{\sqrt{2\kappa }}B\quad \\ {t}^{{\prime} }=\sqrt{2\kappa }t\end{array}\right.$$

we get a generalized equation that does not depend on the PES curvature *κ*

20 $$\left(\begin{array}{r}{\dot{A}}^{{\prime} }\\ {\dot{B}}^{{\prime} }\\ {\dot{\Gamma }}^{{\prime} }\end{array}\right)=\left(\begin{array}{rcl}0&0&1\\ 0&0&-1\\ -1&1&0\end{array}\right)\left(\begin{array}{r}{A}^{{\prime} }\\ {B}^{{\prime} }\\ {\Gamma }^{{\prime} }\end{array}\right)\,.$$

The above equation can be written in a compact notation as

21 $$\dot{{\bf{x}}}={\bf{M}}\cdot {\bf{x}}$$

where the propagation matrix *M* is skew-symmetric. This symmetry imposes that the norm of **x** is conserved

22 $${A}^{{\prime} 2}+{B}^{{\prime} 2}+{\Gamma }^{{\prime} 2}={\rm{const}}\,.$$

In the following, we omit the prime symbol to maintain a cleaner notation and introduce the integer step *n* as *n* = *t*/*dt*. The stability of the methods is investigated by calculating the step transformation matrix **S**(*dt*), which connects the degrees of freedom at time step *n* + 1 with those at step *n*:

23 $${{\bf{x}}}_{n+1}={\bf{S}}(dt){{\bf{x}}}_{n}\,.$$

Iterating Eq. (23), we obtain

24 $${{\bf{x}}}_{n+1}={\bf{S}}{(dt)}^{n}{{\bf{x}}}_{0}\,.$$

The stability condition is achieved if the propagator **S**(*dt*)^*n*^ remains finite for arbitrary large powers *n*. This is equivalent to requiring that all its eigenvalues *λ* are such that ∣*λ*∣≤1. We calculate the step transformation matrices for EE, SIE, and GV schemes. The details of the derivation are reported in Supplementary Section II of the SI. For the EE method we find that

25 $${\lambda }_{\max }=1+2d{t}^{2} \,>\, 1\qquad \forall dt\,,$$

meaning that the EE method is *unconditionally unstable*. The stability condition obtained for the SIE method is instead

26 $$d{t}_{SIE}\le \frac{1}{\omega }\,,$$

where $\omega =\sqrt{\kappa }$ is the frequency of the harmonic oscillator, while for the GV method we obtain

27 $$d{t}_{GV}\le \frac{\sqrt{2}}{\omega }\,.$$

Thus, both the SIE and GV are stable for sufficiently small *dt*, with the GV method having a larger stability range. The integration step of the GV algorithm must thus be shorter than approximately 1/5th of the shortest period of vibrational motion of an atomic system.

### Stochastic formulation

The ensemble averages of the potential energy and its derivatives are multidimensional integrals that are challenging to calculate. One strategy for addressing this issue involves expanding the potential energy in a Taylor series centered at a high-symmetry point of the structure, which allows analytically computing the thermodynamic averages. This approach relies on the analytic knowledge of Gaussian integrals, and it is at the basis of the self-consistent phonon (SCP) approach^35^. The alternative consists in evaluating the integrals through a stochastic Monte Carlo algorithm, as exploited by the SSCHA approach^25^. Here, we introduce a stochastic formulation for the TD-SCHA. The ensemble average of the potential energy on the nuclear density in Eq. (3) can be calculated as:

28 $${\left\langle V\right\rangle }_{{\mathcal{D}}}=\frac{1}{{N}_{c}}\mathop{\sum }\limits_{i=1}^{{N}_{c}}V({\bf{{\mathcal{R}}}}(t)+{\bf{J}}(t)\cdot {{\bf{y}}}_{i}(t))\,,$$

where **y**^*i*^(*t*) are i.i.d. normal random variables, *N*_*c*_ is the number of stochastic configurations, and the subscript ${\mathcal{D}}$ stands for the discrete evaluation of the ensemble average (a more detailed introduction is in Supplementary Section III of the SI). **J**(*t*) is the principal square root (one of many possibilities) of the position-position covariance **A**(*t*):

29 $${J}_{ab}=\sum _{\mu }\sqrt{{\lambda }_{\mu }}{e}_{\mu a}{e}_{\mu b}\,,$$

where *λ*_*μ*_ and *e*_*μ*_ are respectively eigenvalues and eigenvectors of **A**. The TD-SCHA equations only require the averages of the first and second derivatives of the potential. In our formulation, both averages require only the calculation of forces, which can be obtained either from first principles or from surrogate machine-learning force field models. The average of the first derivative of the potential simplifies to

30 $${\left\langle \frac{\partial V}{\partial {R}_{a}}\right\rangle }_{{\mathcal{D}}}=-\frac{1}{{N}_{c}}\mathop{\sum }\limits_{i=1}^{{N}_{c}}{f}_{a}({\bf{\mathcal{R}}}+{\bf{J}}\cdot {{\bf{y}}}_{i})\,,$$

where the Cartesian force component is

31 $${f}_{a}=-\frac{\partial V}{\partial {R}_{a}}\,.$$

(Here, we have omitted the time dependence for clarity in notation). The calculation of the ensemble average of the second derivatives leverages integration by parts to solely utilize the forces^26^

32 $${\left\langle \frac{{\partial }^{2}V}{\partial {R}_{a}\partial {R}_{b}}\right\rangle }_{{\mathcal{D}}}=-\sum _{cd}{A}_{ac}^{-1}\mathop{\sum }\limits_{i=1}^{{N}_{c}}{J}_{cd}\,{y}_{di}{f}_{b}({\bf{\mathcal{R}}}+{\bf{J}}\cdot {{\bf{y}}}_{i})\,.$$

Equation (32) is symmetric in the Cartesian indexes *a* and *b* only in the limit *N*_*c*_ → *∞*. For a finite number of configurations, it must be symmetrized:

33 $${\left\langle \frac{{\partial }^{2}V}{\partial {R}_{a}\partial {R}_{b}}\right\rangle }_{{\mathcal{D}}}^{sym}=\frac{1}{2}{\left\langle \frac{{\partial }^{2}V}{\partial {R}_{a}\partial {R}_{b}}\right\rangle }_{{\mathcal{D}}}+\frac{1}{2}{\left\langle \frac{{\partial }^{2}V}{\partial {R}_{a}\partial {R}_{b}}\right\rangle }_{{\mathcal{D}}}^{T}$$

The stochastic evaluation of these integrals is characterized by Gaussian noise, which decreases as 1/*N*_*c*_. If the random displacements **y**_*i*_ are sampled in uncorrelated way at each time step, this implies the presence of stochastic noise as input in the TD-SCHA differential equations, which can significantly affect their accuracy and stability. As demonstrated in ref. ^24^, the TD-SCHA equations conserve the total energy in the absence of external potentials acting on the system:

34 $$\frac{d}{dt}\sum _{a}\left[\frac{{B}_{aa}+{{\mathcal{P}}}_{a}^{2}}{2}\right]+\frac{d}{dt}\left\langle V\right\rangle =0$$

However, the total energy is conserved only in the limit for *N*_*c*_ → *∞*. We can show that these issues can be fixed by using the same random configurations ${\bar{{\bf{y}}}}_{i}$ in different time step evaluations, which we refer to as correlated sampling. In practice, this means drawing the random configurations ${\bar{{\bf{y}}}}_{i}$ at the first time step and reusing them for the evaluation of ensemble averages at every subsequent time step, rather than generating new configurations at each step. This choice introduces a systematic bias but eliminates the stochastic noise of the ensemble averages of forces and curvatures, making the time evolution smooth. Moreover, we can demonstrate that for one-dimensional problems, energy conservation holds true for any finite number of configurations when a constant ${\bar{{\bf{y}}}}_{i}$ is employed as *dt* → 0 (see Supplementary Section IV of the SI). For higher-dimensional problems, energy conservation remains dependent on the number of configurations due to arbitrariness in the definition of **J**. Nevertheless, the correlated sampling approach drastically improves the energy conservation for a given number of configurations N_*c*_, as shown numerically in the following sections. The possibility of choosing a gauge for **J** that allows for energy conservation independent of the number of configurations in higher-dimensional problems is discussed in the Supplementary Section IV of the SI and will be the subject of future research. The systematic bias introduced by the correlated approach vanishes in the limit *N*_*c*_ → *∞*. Therefore, it can be completely removed by ensuring the convergence of the trajectory with respect to the number of configurations.

### Tests

**Fig. 1:**
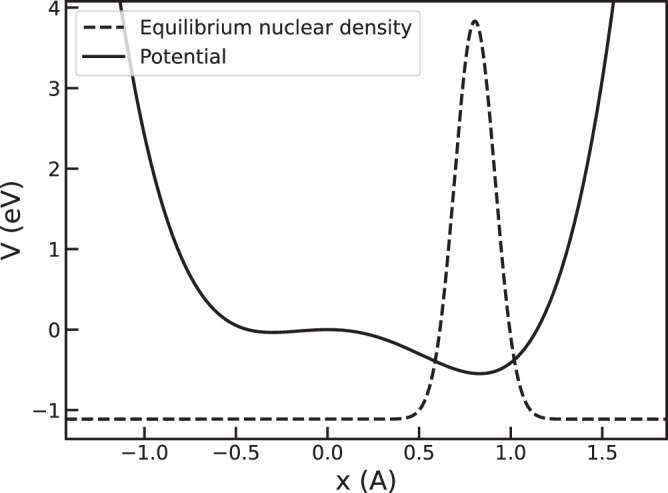
Poltential energy surface of the model. The solid curve represents the potential energy surface of the model, while the dashed Gaussian corresponds to the equilibrium nuclear distribution.

**Fig. 2:**
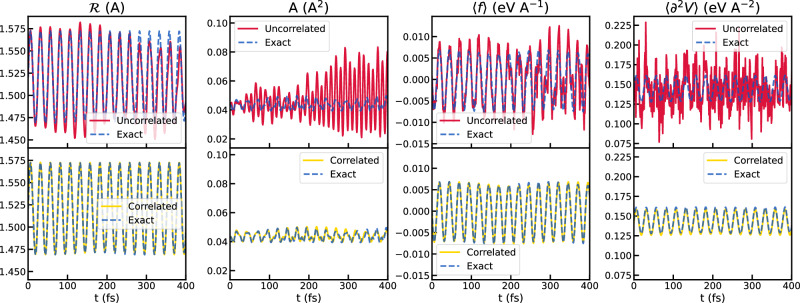
Comparison of uncorrelated and correlated approaches. The panels in the upper row compare the *N*_*c*_ → *∞* solution to the TD-SCHA equations (blue line) with the finite sampling solution using uncorrelated displacement (red line). The lower row of panels compares the exact solution to the TD-SCHA equation with the numerical solution using correlated sampling (yellow line). The quantities represented are (moving from left to right) the position *R*, position-position correlation *A*, average force $\left\langle f\right\rangle$, and average curvature $\left\langle {\partial }^{2}V\right\rangle$. For both the calculations with correlated and uncorrelated displacements, we employed N_c_ = 100 and a time-step of 1 fs.

We test the integration schemes on a one-dimensional model with potential energy

35 $$V(u)=\frac{1}{2}\left(-a{u}^{2}-b{u}^{3}+c{u}^{4}\right)\,,$$

where *a* = 1.00 eV/Å^2^, *b* = 1.00 eV/Å^3^, and *c* = 1.00 eV/Å^4^. The ionic mass is 1 amu. As illustrated in Fig. 1, this potential exhibits two local minima separated by a barrier of approximately 0.58 eV. The initial conditions for the parameters ${\bf{\mathcal{R}}},{\bf{A}},{\bf{B}},{\mathbf{\Gamma }}$ correspond to thermodynamic equilibrium, determined by solving the SSCHA equations^25^ at 100 K. In such equilibrium, the nuclear density is centered at the lowest minimum, with a spread of about 0.5Å due to the ion’s light mass. The initial momentum of the oscillator is set to $\frac{{\mathcal{P}}}{\sqrt{{\rm{m}}}}=0.075\sqrt{{\rm{eV}}}$. First, we assess the accuracy of the stochastic formulation of TD-SCHA introduced in Section “Stochastic formulation”. In particular, we compare the performance of the correlated and uncorrelated approaches for generating random displacements. To achieve this, we solve the TD-SCHA equations using both methods with N_c_= 100 random configurations. The simulation is performed over 400 fs with a time step of 1 fs, employing the GV scheme to integrate Eqs. (8). The results, shown in Fig. 2, are compared with the solution of the TD-SCHA equations in the *N*_*c*_ → *∞* limit, which is numerically obtained by calculating the ensemble averages in Eq. (8) using trapezoidal integration on a dense grid. This is referred to as the “exact” solution. The uncorrelated sampling algorithm quickly deviates from the *N*_*c*_ → *∞* solution. The correlated sampling algorithm, instead, remains stable throughout the dynamics due to the suppression of the stochastic noise across different time steps. This demonstrates the ability of the correlated approach to accurately reproduce the ensemble averages and, consequently, the dynamics.

**Fig. 3:**
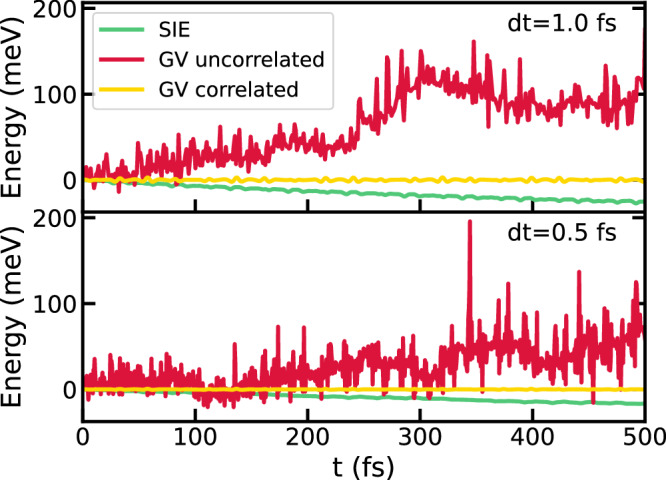
Energy conservation. Energy conservation for different integration schemes and time-step.

**Fig. 4:**
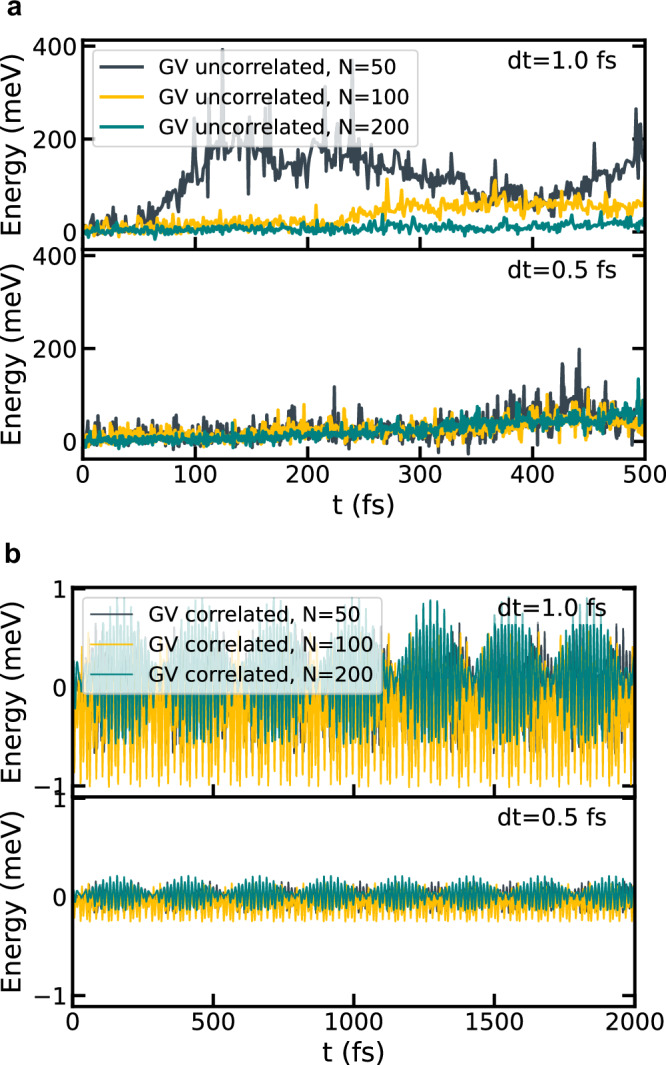
Effect of the number of configurations on energy conservation. **a** Energy conservation of the GV method as a function of the number of configurations *N*_*c*_ in the uncorrelated formulation. **b** Energy conservation in the correlated formulation. Note that the y-scale of the two panels differ by more than two orders of magnitudes.

Next, we evaluate the accuracy of the integration schemes outlined in Section “Numerical integration of TD-SCHA equations” with respect to energy conservation. Figure 3 shows the total energy over time for the GV and SIE schemes using a correlated approach for random displacements, as well as the GV scheme with an uncorrelated approach. The uncorrelated sampling approach fails to conserve energy, although reducing the simulation time step by half partially mitigates this issue. The SIE algorithm with correlated sampling suppresses the energy oscillations, but it suffers from a uniform energy drift that decreases with a reduction in the simulation time step. In contrast, the correlated GV method demonstrates flawless energy conservation for both time steps, exhibiting no energy fluctuations or drift. Finally, we numerically demonstrate that the correlated approach conserves energy independently of the number of configurations. To this end, we perform dynamics simulations for N_c_ = 50, 100, and 200, with the results presented in Fig. 4. For the uncorrelated sampling algorithm, energy conservation improves significantly as the number of configurations increases. In contrast, the correlated sampling approach ensures robust energy conservation, regardless of the number of configurations (see Supplementary Section IV of the SI for the formal proof).

### Dynamics in SrTiO_3_

In this section, we showcase a realistic application of TD-SCHA by investigating the out-of-equilibrium quantum dynamics in SrTiO_3_ (STO) that follows a resonance-exciting short pulse of infrared light. STO is a prototypical quantum paraelectric^36–38^, where nuclear quantum fluctuations suppress the ferroelectric order at low temperatures. STO has been extensively studied due to the emerging phenomena occurring when driven out of equilibrium by strong electric field pulses^39–44^. Notably, irradiating STO with a THz-frequency pulse at low temperatures induces a long-lasting second harmonic generation signal^39,40^, suggesting the occurrence of a light-induced ferroelectric phase transition; however, this interpretation is still debated^45^. Furthermore, recent studies have demonstrated the possibility of transferring energy from lower frequency phonons, pumped by the optical excitation, to higher frequency phonons in an out-of-equilibrium process called upconversion^41^, enabled by the anharmonic coupling between them.

**Fig. 5:**
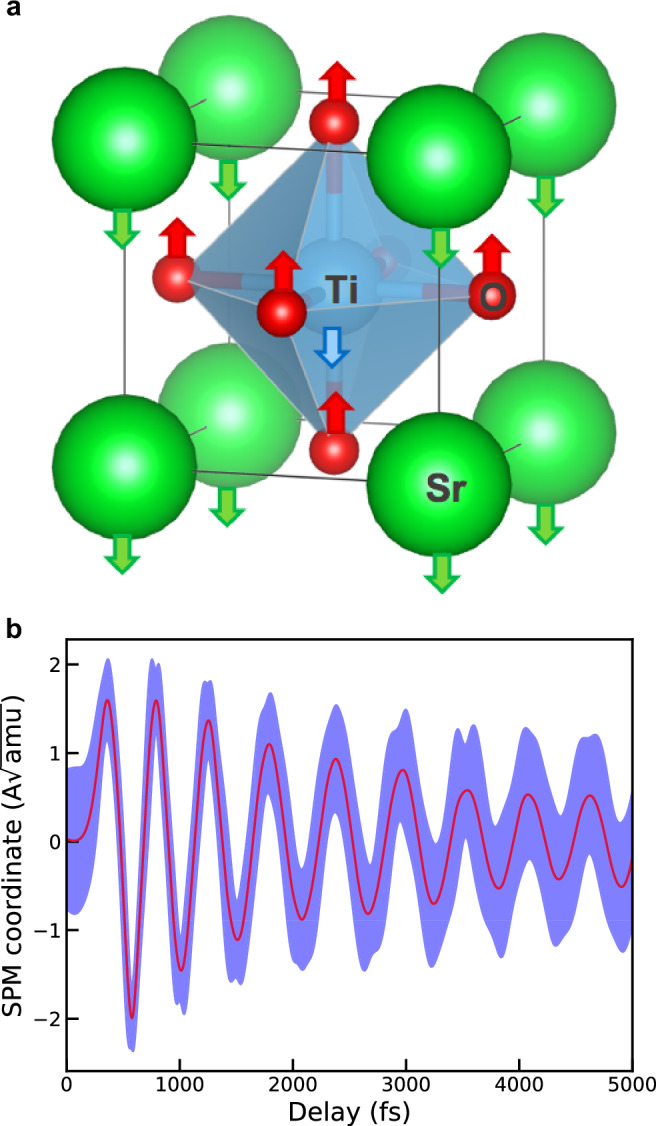
Dynamics in STO. **a** The STO unit cell. The arrows indicate the displacement pattern of the SPM excited resonantly by the THz impulsive pump. **b** Dynamics of the SPM as a function of the time delay after the pulse. The blue area represents the quantum uncertainty, which is comparable to the amplitude of the oscillations, witnessing the importance of nuclear quantum effects in the dynamics.

In our simulation, a 40-atom supercell of STO originally equilibrated at 100 K through a static SSCHA calculation is excited by an infrared pulse with an amplitude of 833 kV/cm, which is resonant with the soft phonon mode (SPM, represented in Fig. 5a of STO. We account for the light-matter interaction in the dipole approximation through the Born effective charges. Details on the coupling with the electric field and the atomic energy landscape calculation are discussed in Method section F. We integrate the TD-SCHA equations using the GV scheme in the correlated formulation, adopting a time step of 1 fs and sampling the potential energy landscape with *N*_*c*_ = 4000. The system, originally in equilibrium at 100 K, interacts at *t* = 0fs with an external pulse of oscillating electric field, triggering a non-equilibrium evolution of the density matrix. Fig. 5b shows the motion of the SPM phonon coordinate

36 $${Q}_{\mu }=\sum _{ax}{e}_{\mu ax}({{\mathcal{R}}}_{ax}-{{\mathcal{R}}}_{ax}^{eq})$$

as a function of the time delay after the pulse. Here ${{\mathcal{R}}}_{ax}^{eq}$ represents the equilibrium centroid position of the atom *a* in the direction *x*, and *e*_*μax*_ is the equilibirum eigenvector of the soft phonon mode *μ*. The irradiation of STO with resonant pulses drives large oscillations of the SPM, which slowly decay due to the interaction with other phonon modes. The blue area in the figure corresponds to the quantum uncertainty in the position of the SPM. It is computed as $\pm \sqrt{{A}_{\mu \mu }}$, which is equal to $\sqrt{\left\langle {Q}_{\mu }^{2}\right\rangle }$. The large extent of this uncertainty relative to the motion of *Q*_*μ*_ highlights the fundamental importance of quantum effects in the dynamics of STO. An extensive discussion of the relevance of the simulation for the physics of STO goes beyond the scope of this work, and is subject of a separate publication^46^.

In conclusion, we introduced the first approach to simulate nonequilibrium quantum nuclear dynamics using the TD-SCHA. We derived an integration scheme, the Generalized Verlet, which allows for the evolution of the equations with an error of ${\mathcal{O}}(d{t}^{3})$, demonstrating that its conditional stability is consistent and of the same order as the Nyquist sampling rate. Additionally, we introduced a stochastic formulation of the TD-SCHA, which enables efficient calculation of ensemble averages while ensuring the stability of the evolution. Finally, we showcased the method’s potential, proving it is well-suited for simulating quantum nonequilibrium processes in pump-probe setups on the scale of hundreds of atoms.

## Methods

### Machine learned potential

The TD-SCHA simulations on STO utilize machine-learned potentials (MLP) to model the potential energy surface. We opted for FLARE^47^ for its active learning capabilities, enabling efficient data generation and rapid inference times^48^. Through active learning, we explored various temperatures and volumes to develop a broadly applicable potential.

Simulations were conducted at 100, 300, and 500 K, each for 200 ps, at the DFT-relaxed lattice parameter, as well as at ±2% strain, amounting to a total of 1.8 ns of dynamics. A timestep of 2 fs and a thermostat damping time of 200 fs were employed, utilizing the default Nosé-Hoover thermostat in LAMMPS^49^.

DFT calculations were performed at the PBE level of theory using Quantum ESPRESSO^50^. We used a plane-wave cutoff of 80 Ry and a k-point grid of 6 × 6 × 8 for the 20-atom cell, adopting the pseudopotentials recommended by the SSSP efficiency library^51^.

### Interaction with the electric field

The forces on the atom *i* by the electric field is obtained as

37 $${{\bf{f}}}_{i}=\frac{1}{{\varepsilon }_{eff}}\,{{\bf{Z}}}_{i}\cdot {\bf{{\mathcal{E}}}}\,.$$

Here ${\bf{{\mathcal{E}}}}$ is the external electric field, *ε*_*eff*_ is the dielectric constant and **Z**_*i*_ are the Born effective charge tensors. The effective charges are computed through DFPT, using the same parameters as above. Their value for the different atomic species is reported in Table 1.

**Table 1:** Born effective charges for the cubic STO unitcell, computed through DFPT

	${{\rm{Z}}}_{{\rm{xx}}}^{* }$	${{\rm{Z}}}_{{\rm{yy}}}^{* }$	${{\rm{Z}}}_{{\rm{zz}}}^{* }$
Ti	7.338	7.338	7.338
Sr	2.549	2.549	2.549
O_1_	−2.024	−5.845	−2.024
O_2_	−2.024	−2.024	−5.845
O_3_	−5.845	−2.024	−2.024

Here we employ the screening model proposed in refs. ^42,52^

38 $${\varepsilon }_{eff}=\frac{1+\sqrt{{\varepsilon }_{DFPT}}}{2}\,,$$

with *ε*_*DFPT*_ = 6.31.

## Supplementary information

Supplementary Materials

## Data Availability

No datasets were generated or analyzed during the current study.
