# Supplementary material for: Atomistic simulations of out-of-equilibrium quantum nuclear dynamics
Source: NPJ Comput Mater. 2025 Apr 16;11(1):102. doi: 10.1038/s41524-025-01588-4 (PMC12003180; doi:10.1038/s41524-025-01588-4)
Supplement: Supplementary file 1 — Supplementary Materials [file 41524_2025_1588_MOESM1_ESM.pdf]

Supplementary Materials for

Atomistic simulations of out-of-equilibrium quantum nuclear dynamics

Francesco Libbi *et al.*

I. NOTES ON THE WIGNER FORMULATION OF TD-SCHA

The nuclear density introduced in Eq. 3 of the main text corresponds to the most general Gaussian form in the positions $\mathbf{R}$ and momenta $\mathbf{P}$ variables. It is parametrized by the vectors $\boldsymbol{\mathcal{R}}$ and $\boldsymbol{\mathcal{P}}$ and the matrices $\mathbf{A}$, $\mathbf{B}$, and $\boldsymbol{\Gamma}$. The physical meaning of these parameters is immediately clear after noting that

$$\boldsymbol{\mathcal{R}}(t) = \langle \mathbf{R} \rangle (t) \ , \tag{1}$$

$$\boldsymbol{\mathcal{P}}(t) = \langle \mathbf{P} \rangle (t) \ , \tag{2}$$

$$A_{ij}(t) = \langle \delta R_i \delta R_j \rangle \ , \tag{3}$$

$$B_{ij}(t) = \langle \delta P_i \delta P_j \rangle \ , \tag{4}$$

$$\Gamma_{ij}(t) = \langle \delta R_i \delta P_j \rangle \ . \tag{5}$$

These properties can be easily shown using the definition of ensemble averages given in Eq. 2 of the main text. According to Eqs. 1 and 2, the parameters $\boldsymbol{\mathcal{R}}$ and $\boldsymbol{\mathcal{P}}$ correspond to the expected values for the positions and momenta, which are analogous to the classical positions and momenta. The variables $\mathbf{A}$, $\mathbf{B}$ and $\boldsymbol{\Gamma}$, instead, correspond to the position-position, momentum-momentum and position-momentum correlation matrices, as suggested by Eqs. 3-5.
If an observable is a function of only the position $O(\mathbf{R})$, the momenta degrees of freedom can be integrated out in Eq. 2 of the main text. After some tedious algebra, it is possible to show that in such case

$$\langle O \rangle = \int O(\boldsymbol{\mathcal{R}}(t) + \delta\mathbf{R}) \sqrt{\frac{1}{(2\pi)^{3N}\det A}} e^{-\frac{1}{2}\delta\mathbf{R}\cdot\mathbf{A}^{-1}(t)\cdot\delta\mathbf{R}} \ d(\delta\mathbf{R}) \ . \tag{6}$$

We can clearly see that $\langle O \rangle (t)$ is a function of $\boldsymbol{\mathcal{R}}$ and $\mathbf{A}$ only.

II. NUMERICAL STABILITY OF INTEGRATION SCHEMES

First of all, we prove an identity for the central finite difference approximation of a derivative. We first write the Taylor expansion of a function F up to the third order centered at t:

$$F_{t+dt} = F_t + F'_t dt + \frac{1}{2} F''_t dt^2 + \mathcal{O}(dt^3) \tag{7}$$

we can write the same expression by centering it in $t + dt$,

$$F_t = F_{t+dt} - F'_{t+dt} dt + \frac{1}{2} F''_{t+dt} dt^2 + \mathcal{O}(dt^3) \tag{8}$$

Adding up these equations we get

$$F_{t+dt} = F_t + \frac{1}{2}(F'_t + F'_{t+dt})dt + \frac{1}{2}(F''_t - F''_{t+dt})dt^2 + \mathcal{O}(dt^3) \ . \tag{9}$$

However, from the following equation

$$F''_{t+dt} = F''_t + F'''_t dt + \mathcal{O}(dt^2) \tag{10}$$

we get

$$(F''_{t+dt} - F''_t)dt^2 = F'''_t dt^3 + \mathcal{O}(dt^4) \tag{11}$$

leading to the result

$$F_{t+dt} = F_t + \frac{1}{2}(F'_t + F'_{t+dt})dt + \mathcal{O}(dt^3) \ , \tag{12}$$

which corresponds to Eq. 13 of the main text. This equation indicates that we can evolve a function F with accuracy up to third-order by utilizing the average of the derivative at time t and that at time $t+dt$. Eq. 12 can be employed to derive the Verlet algorithm:

$$\begin{cases} \boldsymbol{\mathcal{P}}_{t+dt} = \boldsymbol{\mathcal{P}}_t + \frac{1}{2}\Big(\langle \mathbf{f} \rangle_t + \langle \mathbf{f} \rangle_{t+dt}\Big)dt + \mathcal{O}(dt^3) \\ \boldsymbol{\mathcal{R}}_{t+dt} = \boldsymbol{\mathcal{R}}_t + \boldsymbol{\mathcal{P}}_t dt + \frac{1}{2}\langle \mathbf{f} \rangle_t dt^2 + \mathcal{O}(dt^3) \end{cases} . \tag{13}$$

In the following, we study the stability of the explicit Euler, semi-implicit Euler and generalized Verlet methods.

1. Explicit Euler method

At each step of the explicit Euler method, the variables are updated according to the following rule:

$$\mathbf{x}_{n+1} = \begin{pmatrix} 1 & 0 & dt \\ 0 & 1 & -dt \\ -dt & dt & 1 \end{pmatrix} \mathbf{x}_n = \mathbf{S}(dt)\mathbf{x}_n \tag{14}$$

leading to

$$\mathbf{x}_{n+1} = \mathbf{S}(dt)^n \mathbf{x}_0 \tag{15}$$

The exponentiation of such a matrix requires the calculation of its eigenvalue, which are

$$\begin{cases} \lambda_1 = 1 \\ \lambda_2 = 1 - i\sqrt{2}dt \\ \lambda_3 = 1 + i\sqrt{2}dt \end{cases} \tag{16}$$

The stability condition for preventing divergence of the power sequence in Eq. 15 is

$$|1 \pm i\sqrt{2}dt| \leq 1 \tag{17}$$

which is never satisfied. The explicit Euler method is thus *unconditionally unstable.*

2. Semi-implicit Euler method

At each step, first Γ is updated:

$$\Gamma_{n+1} = \Gamma_n + (B_n - A_n)dt \tag{18}$$

and then A and B

$$\begin{cases} A_{n+1} = A_n + \Gamma_{n+1}dt \\ B_{n+1} = B_n - \Gamma_{n+1}dt \end{cases} \tag{19}$$

Substituting Eq. 18 into 19, we obtain

$$\begin{cases} A_{n+1} = A_n + \Gamma_n dt + (B_n - A_n)dt^2 \\ B_{n+1} = B_n - \Gamma_n dt - (B_n - A_n)dt^2 \end{cases} \tag{20}$$

or equivalently

$$\begin{pmatrix} A_{n+1} \\ B_{n+1} \\ \Gamma_{n+1} \end{pmatrix} = \begin{pmatrix} 1 - dt^2 & dt^2 & dt \\ dt^2 & 1 - dt^2 & -dt \\ -dt & dt & 1 \end{pmatrix} \begin{pmatrix} A_n \\ B_n \\ \Gamma_n \end{pmatrix} . \tag{21}$$

The eigenvalues of the step matrix are

$$\begin{cases} \lambda_1 = 1 \\ \lambda_2 = 1 - dt^2 - dt\sqrt{dt^2 - 2} \\ \lambda_3 = 1 - dt^2 + dt\sqrt{dt^2 - 2} \end{cases} \tag{22}$$

The eigenvalues λ_2, λ_3 are real for $dt \geq \sqrt{2}$, or complex conjugated otherwise. We easily note that

$$\lambda_2 < -1 \qquad \forall dt \geq \sqrt{2} \tag{23}$$

meaning that this method is *unstable* in such range. For $dt < \sqrt{2}$, $\lambda_{2,3}$ are complex, with modulus

$$|\lambda_{2,3}|^2 = (1 - dt^2)^2 + 2dt^2 - dt^4 = 1 \tag{24}$$

therefore the method is *stable*. Remembering the definition 19 in the main text, the stability condition for this method is

$$t \leq \frac{1}{\sqrt{\kappa}} = \frac{1}{\omega} \tag{25}$$

where ω is the frequency of the harmonic oscillator (we remind that $\kappa = \frac{1}{m}\frac{\partial^2 V}{\partial R^2}$ due to the mass rescaling convention adopted).

3. Generalized Verlet

The same analylisis can be performed on the GV algorithm. In this case, the variables are updated according to

$$\begin{cases} A_{n+1} = A_n + \Gamma_n dt + \frac{1}{2}(B_n - A_n)dt^2 \\ B_{n+1} = B_n - \frac{1}{2}(\Gamma_n + \Gamma_{n+1})dt \\ \Gamma_{n+1} = \Gamma_n + \frac{1}{2}(B_n - A_n + B_{n+1} - A_{n+1})dt \end{cases} \tag{26}$$

After some tedious algebra, it is possible to write explicitly the transformation in matrix form as

$$\begin{pmatrix} A_{n+1} \\ B_{n+1} \\ \Gamma_{n+1} \end{pmatrix} = \begin{pmatrix} 1 - \frac{dt^2}{2} & \frac{dt^2}{2} & dt \\ \frac{-dt^4+4dt^2}{2dt^2+8} & \frac{dt^4-2dt^2+8}{2dt^2+8} & \frac{dt^3-4dt}{dt^2+4} \\ \frac{dt^3-4dt}{dt^2+4} & \frac{-dt^3+4dt}{dt^2+4} & \frac{-3dt^2+4}{dt^2+4} \end{pmatrix} \begin{pmatrix} A_n \\ B_n \\ \Gamma_n \end{pmatrix} , \tag{27}$$

with eigenvalues

$$\begin{cases} \lambda_1 = 1 \\ \lambda_2 = \frac{4-3dt^2-2\sqrt{2}dt\sqrt{dt^2-4}}{dt^2+4} \\ \lambda_3 = \frac{4-3dt^2+2\sqrt{2}dt\sqrt{dt^2-4}}{dt^2+4} \end{cases} . \tag{28}$$

The eigenvalues are complex for $dt < 2$, with modulus

$$|\lambda_{2,3}|^2 = 1 , \tag{29}$$

and the method is stable, whereas for $dt > 2$

$$\lambda_2 < -1 , \tag{30}$$

thus the method is unstable. To conclude, the stability condition for such an algorithm is

$$dt < \sqrt{\frac{2}{\kappa}} = \frac{\sqrt{2}}{\omega} . \tag{31}$$

The stability range is thus larger than that of the SIE.

III. IMPORTANCE SAMPLING

In the following section we define some properties which will be useful for the development of the theory reported in the main text. The ensemble average of the potential energy at time t is given by

$$\langle V \rangle = \int V(\boldsymbol{\mathcal{R}}(t) + \delta\mathbf{R}) \sqrt{\frac{1}{(2\pi)^{3N}\det A}} e^{-\frac{1}{2}\delta\mathbf{R}\cdot\mathbf{A}^{-1}(t)\cdot\delta\mathbf{R}} \, d(\delta\mathbf{R}) \tag{32}$$

where the integration is carried over the variables δR_a whose number is $3N$. We now perform a change of variables which is meant to turn the Gaussian in Eq. 32 into a normal distribution. We start from the modal decomposition of the matrix A

$$A_{ab} = \sum_\mu \lambda_\mu e_{\mu a} e_{\mu b} \ , \tag{33}$$

and express the displacement δR_a in normal coordinates:

$$\delta R_a = \sum_b J_{ab} y_b \ . \tag{34}$$

The determinant of the Jacobian matrix is

$$\det\, J = \prod_\mu \sqrt{\lambda_\mu} = \sqrt{\det\, A} \ . \tag{35}$$

The change of variables of Eq. 34 turns the integral 32 into

$$\langle V \rangle = \int V(\mathcal{R}_a(t) + \sum_b J_{ab}(t) y_b) \prod_b \frac{e^{-\frac{1}{2}y_b^2}}{\sqrt{2\pi}} dy_b \ . \tag{36}$$

Eq. 36 provides the starting point for the stochastic calculation of the ensemble averages, expressed in Eq. 28 of the main text.

IV. ENERGY CONSERVATION

Here, we outline the main steps introduced in Ref.[1] to derive energy conservation for infinitely many configurations. We then show the conditions under which such conservation holds regardless of the number of configurations, which represents the result of the current work. The time derivative of the quantum kinetic energy reads

$$\frac{d}{dt} \sum_i \Big\langle \frac{P_i^2}{2} \Big\rangle = \frac{d}{dt} \frac{\sum_i \mathcal{P}_i^2 + \mathrm{Tr}\ \mathbf{B}}{2} = \sum_i \Big(\mathcal{P}_i \dot{\mathcal{P}}_i + \frac{\dot{B}_{ii}}{2} \Big) \tag{37}$$

Combining the first and second of Eqs. 8 of the main text, we obtain

$$\sum_i \mathcal{P}_i \dot{\mathcal{P}}_i = - \sum_i \Big\langle \frac{\partial V}{\partial R_i} \Big\rangle \dot{\mathcal{R}}_i \ , \tag{38}$$

while the third and fourth of Eqs. 8 give

$$\sum_i \frac{\dot{B}_{ii}}{2} = -\frac{1}{2} \sum_{ij} \Big\langle \frac{\partial^2 V}{\partial R_i \partial R_j} \Big\rangle \dot{A}_{ij} \ . \tag{39}$$

Importantly, these equations also hold when the discrete expressions for forces (Eq. 30 of the main text) and curvatures (Eq. 33 of the main text) are used to drive the dynamics

$$\sum_i \mathcal{P}_i \dot{\mathcal{P}}_i = - \sum_i \Big\langle \frac{\partial V}{\partial R_i} \Big\rangle_{\mathcal{D}} \dot{\mathcal{R}}_i \ , \tag{40}$$

$$\sum_i \frac{\dot{B}_{ii}}{2} = -\frac{1}{2} \sum_{ij} \Big\langle \frac{\partial^2 V}{\partial R_i \partial R_j} \Big\rangle_{\mathcal{D}} \dot{A}_{ij} \ . \tag{41}$$

Following Ref.[1], it is possible to show that

$$\Big\langle \frac{\partial V}{\partial R_i} \Big\rangle = \frac{\partial \langle V \rangle}{\partial \mathcal{R}_i} \tag{42}$$

and

$$\frac{1}{2}\left\langle \frac{\partial^2 V}{\partial R_i R_j} \right\rangle = \frac{\partial \langle V \rangle}{\partial \mathcal{A}_{ij}} \ . \tag{43}$$

Inserting Eqs. 42 and 43 into Eq. 37 we obtain

$$\frac{1}{2}\frac{d}{dt}\Big(\sum_i \mathcal{P}_i^2 + \mathrm{Tr}\ \mathbf{B}\Big) = -\frac{\partial \langle V \rangle}{\partial \mathcal{R}_i}\dot{\mathcal{R}}_i - \frac{\partial \langle V \rangle}{\partial \mathcal{A}_{ij}}\dot{A}_{ij} = -\frac{d}{dt}\langle V \rangle \ , \tag{44}$$

This proves the conservation of energy in absence of external forces. However, for this energy conservation to hold for any configuration, we need to demonstrate that Eqs. 42 and 43 also hold for the discrete expression of forces and curvatures, namely:

$$\left\langle \frac{\partial V}{\partial R_i} \right\rangle_{\mathcal{D}} = \frac{\partial \langle V \rangle_{\mathcal{D}}}{\partial \mathcal{R}_i} \tag{45}$$

and

$$\frac{1}{2}\left\langle \frac{\partial^2 V}{\partial R_i R_j} \right\rangle_{\mathcal{D}} = \frac{\partial \langle V \rangle_{\mathcal{D}}}{\partial \mathcal{A}_{ij}} \ . \tag{46}$$

Equation 45 can be easily derived by applying the chain rule to differentiate Equation 28 of the main text:

$$\left\langle \frac{\partial V}{\partial R_a} \right\rangle_{\mathcal{D}} = -\frac{1}{N_c}\sum_{i=1}^{N_c} f_a(\boldsymbol{\mathcal{R}} + \mathbf{J}\cdot\mathbf{y}_i) = \frac{1}{N_c}\sum_{i=1}^{N_c}\frac{\partial V}{\partial \mathcal{R}_a}(\boldsymbol{\mathcal{R}} + \mathbf{J}\cdot\mathbf{y}_i) = \frac{\partial \langle V \rangle_{\mathcal{D}}}{\partial \mathcal{R}_a} \ , \tag{47}$$

The derivation of Eq. 46 is more complex, and does not hold in the most general case. From Eqs. 32 and 33 of the main text, we know that

$$\left\langle \frac{\partial^2 V}{\partial R_a \partial R_b} \right\rangle_{\mathcal{D}}^{sym} = -\frac{1}{2}\sum_{cd} A^{-1}_{ac}\sum_{i=1}^{N} J_{cd}y_{di}f_b(\boldsymbol{\mathcal{R}} + \mathbf{J}\cdot\mathbf{y}_i) \ -\frac{1}{2}\sum_{cd} A^{-1}_{bc}\sum_{i=1}^{N} J_{cd}y_{di}f_a(\boldsymbol{\mathcal{R}} + \mathbf{J}\cdot\mathbf{y}_i) \ . \tag{48}$$

At the same time, the derivative of $\langle V \rangle_{\mathcal{D}}$ with the respect to the element A_{ab} is

$$\frac{\partial}{\partial A_{ab}}\langle V \rangle_{\mathcal{D}} = -\frac{1}{N_c}\sum_{cd}\sum_{i=1}^{N_c} f_c(\boldsymbol{\mathcal{R}} + \mathbf{J}\cdot\mathbf{y}_i)\frac{\partial J_{cd}}{\partial A_{ab}}y_d \tag{49}$$

In order to prove Eq. 46, it would be enough to show that

$$\frac{\partial J_{cd}}{\partial A_{ab}} = \frac{1}{4}\delta_{ac}J^{-1}_{bd} + \frac{1}{4}\delta_{bc}J^{-1}_{ad} \ . \tag{50}$$

In one dimensional problems, identity 50 reduces to

$$\frac{\partial J}{\partial A} = \frac{1}{2\sqrt{A}} \ , \tag{51}$$

which is satisfied since $J = \sqrt{A}$. Therefore, in one dimensional problems, the conservation of energy holds independently on the number of configurations, provided that the time step is reasonably small.
For higher dimensional problems, the validity of the identity 50 depends on the definition of $\mathbf{J}$. In fact, there are infinite many ways to obtain the square root of a symmetric matrix, defined as the matrix $\mathbf{J}$ such that

$$\mathbf{A} = \mathbf{J}\mathbf{J}^{\mathrm{T}} \ . \tag{52}$$

This is because multiplying the matrix $\mathbf{J}$ by any orthogonal matrix $\mathbf{O}$ results in a matrix that still satisfies Eq. 52. The definition of $\mathbf{J}$ in Eq. 29 of the main text does not satisfy the identity 50. In fact, using the rules derived in Appendix 5 of Ref.[2], we easily obtain

$$\sum_{ab}\frac{\partial J_{cd}}{\partial A_{ab}}\dot{A}_{ab} = \sum_{ab}\sum_{\mu\nu}\frac{e_{a\mu}e_{b\nu}e_{c\mu}e_{d\nu}}{\sqrt{\lambda_\mu} + \sqrt{\lambda_\nu}}\dot{A}_{ab} \ . \tag{53}$$

The problem of finding an expression for $\mathbf{J}$ that satisfies identity 50 is very important and will be the subject of future research efforts.

---

[1] A. Siciliano, L. Monacelli, G. Caldarelli, and F. Mauri, Phys. Rev. B **107**, 174307 (2023).
[2] R. Bianco, I. Errea, L. Paulatto, M. Calandra, and F. Mauri, Phys. Rev. B **96**, 014111 (2017).
